# The complete mitochondrial genome of *Gibbovalva kobusi* (Lepidoptera: Gracillariidae)

**DOI:** 10.1080/23802359.2019.1644550

**Published:** 2019-07-29

**Authors:** Lu Chen, Cheng-Qing Liao, Xing Wang, Shao-Xian Tang

**Affiliations:** a College of Plant Protection, Hunan Agricultural University, Changsha, Hunan, China;; b Hunan Provincial Key Laboratory for Biology and Control of Plant Diseases and Insect Pests, Hunan Agricultural University, Changsha, Hunan, China;; c College of Information Science and Technology, Hunan Agricultural University, Changsha, Hunan, China

**Keywords:** *Gibbovalva kobusi*, mitochondrial genome, evolutionary position

## Abstract

In this study, the complete mitochondrial genome (mitogenome) of *Gibbovalva kobusi* was at first sequenced by high-throughput sequencing. As a circular DNA molecule, the complete mitogenome is 15,717 bp in length (GeneBank accession number: MK956103) and consists of 13 protein-coding genes (PCGs), 22 transfer RNA (tRNA) genes, 2 ribosomal RNA (rRNA) genes, and an AT-rich region. The nucleotide composition is A (41.0%), C (11.6%), G (7.9%), and T (39.5%). Based on the sequences of complete mitogenome from 11 species as ingroups and 3 superfamily Tineoidea species as outgroups, the phylogenetic trees were constructed. The family Gracilariidae as a monophyletic clade is strongly supported by the bootstrap value of 100%.

*Gibbovalva kobusi* belongs to Acrocercopinae in the family Gracilariidae (Lepidoptera: Gracillarioidea) (Kawahara et al. 2017) and mainly damages the leaves of the families Lauraceae and Magnoliaceae (Bai and Li 2008; Xu et al. 2014; Bai et al. 2016; De Prins and De Prins 2018). In this study, *G. kobusi*, while mining for *Magnolia officinalis* and *Liriodendron chinense* (Magnoliaceae), was collected from Tianpingshan Mountain (29°N, 110°E, elevation of 1332 m) in Badagongshan National Nature Reserve (Sangzhi County, Zhangjiajie City, Hunan Province) and its complete mitochondrial genome (mitogenome) was reported for the first time. All specimens and the genomic DNA were deposited at the Insect Museum of Hunan Agricultural University, Changsha City, Hunan Province, China. We gained the complete mitogenome of *G. kobusi* and constructed a phylogenetic tree for understanding its phylogenetic position in the superfamily Gracillarioidea and relationships with other closely related groups.

The complete genome DNA was extracted from the larvae (collection number: LCQ006) by using TaKaRa MiniBEST Universal Genomic DNA Extraction Kit Ver.5.0 (Shiga Prefecture, Kusatsu City, Japan) and was sequenced by using high-throughput sequencing in Berry Genomics Corporation. The assembly of sequence used IDBA in server. The gene annotation was accomplished with ORF finder (https://www.ncbi.nlm.nih.gov/orffinder/) and MITOS web server (http://mitos.bioinf.uni-leipzig.de/index.py) (Bernt et al. 2013). To construct the phylogenetic tree, the complete mitogenomes of 11 species in the superfamilies Gracillarioidea, Yponomeutoidea, and Gelechioidea and 3 species of the superfamily Tineoidea as outgroups were used in the analyses. The maximum likelihood (ML) method was performed by MEGA 10 and the bootstrap analysis was set as 1000 pseudoreplicates.

The complete mitogenome of *G. kobusi* is a circular DNA molecule of 15,717 bp in length (GeneBank accession number: MK956103) and consists of 13 protein-coding genes (PCGs), 22 transfer RNA (tRNA) genes, 2 ribosomal RNA (rRNA) genes, and an AT-rich region, in which 23 genes are transcribed on the J strand and the remaining 14 are transcribed on the N strand. The nucleotide composition is A (41.0%), C (11.6%), G (7.9%), T (39.5%), and the AT nucleotide content is 80.5%. There were 469 bp intergenic nucleotides that were dispersed in between 12 pairs of neighboring genes with their length varying from 1 to 319 bp. The length of the A + T rich region that was located between *rrnS* and the *trnM* was 503 bp. The phylogenetic tree based on sequences of the complete mitogenome was shown in Figure 1. The relationship within this superfamily Gracilarioidea that is a monophyletic clade is strongly supported by the bootstrap value of 100% and as a sister group of the superfamily Yponomeutoidea is also supported by the bootstrap value of 91%.

**Figure 1.**
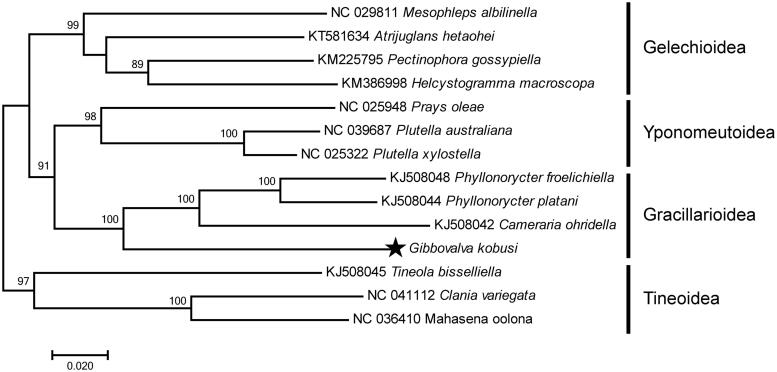
Maximum-likelihood tree of evolutionary relationships *Gibbovalva kobusi* based on the complete mitogenomes of 14 Lepidopteran moths. All the species’accession numbers in this study are listed as below: *Mesophleps albilinella* NC 029811, *Atrijuglans hetaohei* KT581634, *Pectinophora gossypiella* KM225795, *Helcystogramma macroscopa* KM386998, *Prays oleae* NC 025948, *Plutella australiana* NC 039687, *Plutella xylostella* NC 025322, *Phyllonorycter froelichiella* KJ5008048, *Phyllonorycter platani* KJ508044, *Cameraria ohridella* KJ508042, *Tineola bisselliella* KJ508045, *Clania veriegata* NC 041112, *Mahasena oolona*, NC 036410.
